# Macropinocytosis: a pathway to protozoan infection

**DOI:** 10.3389/fphys.2015.00106

**Published:** 2015-04-09

**Authors:** Tecia M. U. de Carvalho, Emile S. Barrias, Wanderley de Souza

**Affiliations:** ^1^Laboratório de Ultraestrutura Celular Hertha Meyer, Instituto de Biofísica Carlos Chagas Filho, Centro de Ciência da Saúde, Universidade Federal do Rio de JaneiroRio de Janeiro, Brazil; ^2^Instituto Nacional de Ciência e Tecnologia em Biologia Estrutural e Bioimagens e Centro Nacional de Bioimagens-CENABIO, Universidade Federal do Rio de JaneiroRio de Janeiro, Brazil; ^3^Instituto Nacional de Metrologia, Qualidade e Tecnologia—InmetroRio de Janeiro, Brazil

**Keywords:** macropinocytosis, protozoa, actin filaments

## Abstract

Among the various endocytic mechanisms in mammalian cells, macropinocytosis involves internalization of large amounts of plasma membrane together with extracellular medium, leading to macropinosome formation. These structures are formed when plasma membrane ruffles are assembled after actin filament rearrangement. In dendritic cells, macropinocytosis has been reported to play a role in antigen presentation. Several intracellular pathogens are internalized by host cells via multiple endocytic pathways and macropinocytosis has been described as an important entry site for various organisms. Some bacteria, such as *Legionella pneumophila*, as well as various viruses, use this pathway to penetrate and subvert host cells. Some protozoa, which are larger than bacteria and virus, can also use this pathway to invade host cells. As macropinocytosis is characterized by the formation of large uncoated vacuoles and is triggered by various signaling pathways, which is similar to what occurs during the formation of the majority of parasitophorous vacuoles, it is believed that this phenomenon may be more widely used by parasites than is currently appreciated. Here we review protozoa host cell invasion via macropinocytosis.

The set of mechanisms known as endocytosis can be described as those processes that allow communication between intracellular and extracellular environments, where molecules exposed to the outer face of the plasma membrane play an important role. However, the mechanisms regulating endocytosis are also dependent upon events that take place on the cytoplasmic face of the plasma membrane, as well as upon cytoskeletal components associated to the membrane. In addition, as consequence of the activation of a series of intracellular signaling cascades, the process of endocytosis interfere with the control of basic cell phenomena, such as the control of cell migration, cell division, cell-to-cell interaction, and antigen presentation (Hoeller et al., 2005). In recent years several studies have indicated that macropinocytosis is also used by various pathogens (e.g., viruses, bacteria, and protozoa) to gain access to different cell types (Marsh and Helenius, 2006).

Several groups have analyzed in detail the endocytic processes using various approaches, allowing characterization of the size of the endocytic vesicle, the nature of the cargo and the mechanisms of vesicle formation (Veiga and Cossart, 2006). Currently, endocytic events are divided into the follow routes: classical endocytosis (clathrin-mediated); caveolin-dependent endocytosis 1 (caveolae); endocytic type CLIC/GEEC; ARF6-dependent endocytosis; flotilin dependent endocytosis; phagocytosis; and macropinocytosis (Doherty and McMahon, 2009).

The macropinocytosis pathway involves internalization of a large area of the plasma membrane together with significant amounts of extracellular fluid. This process occurs when membrane projections fuse with each other, generating a large and irregular vesicle (>1 μm) known as the macropinosome. These structures are formed from plasma membrane “ruffles,” which are generated following the rearrangement of cortical actin filaments (Johannes and Lamaze, 2002).

Normally, macropinocytosis events are initiated from an external stimulus. These stimuli may be growth factors that lead to activation of tyrosine kinase type receptors that subsequently trigger the activation of a signaling cascade that culminates in the remodeling of the actin cytoskeleton and subsequent formation of membrane ruffles (Mercer and Helenius, 2009). In this activation process proteins belonging to the Ras superfamily of GTPases play a fundamental role. Proteins of this superfamily, when activated by a tyrosine kinase receptor, trigger three parallel signaling pathways involving Ras-related C3 botulinum toxin substrate 1 (Rac1), Rab5, ARF6, and phosphoinositide 3-kinase (PI3K) (Bar-Sagi et al., 1987). These pathways play important roles in the formation of membrane ruffles, as well as in macropinosome traffic. Inhibitors of phosphoinositide 3-kinases (PI3Ks), which generate PI(3,4,5)P3 from PI(4,5)P2, impair macropinosome formation since the transient and sequential emergence of phosphoinositides PI(3,4,5)P3 and PI(3,4)P2 in the membrane ruffles are essential for macropinocytosis (Araki et al., 1996; Maekawa et al., 2014). Rac 1 and ARF6, both proteins involved in actin cytoskeleton rearrangement, are involved in ruffle formation. ARF6 can activate Rac 1 to promote the formation of a macropinosome (reviewed by Donaldson et al., 2009). Rac 1 activation and deactivation has a crucial role in macropinocytosis (Fujii et al., 2013). Once activated, Rac 1 triggers effector proteins that leads to actin polymerization, filaments stabilization, and the effector-dependent myosin contraction (Ridley and Hall, 1996; Mercer and Helenius, 2009). Rab5, a Rab GTPase protein and well-known molecular marker of the early endosome, and an increased expression of Rab5 (together with an active form of Ras) promotes the formation of circular ruffles. This protein relocates along the ruffle regions with RN-Tre (related protein N-terminal threonine). RN-Tre and the Rab5 effector protein, GAP, interact with F-actin to promote crosslinking between ruffle regions and actin filaments (Lanzetti and Fiore, 2008). In addition, one of Rab5 effectors, known as Rabankirin 5, promotes macropinocytosis and has been determined as a molecular marker of this event (Schnatwinkel et al., 2004). This protein is also responsible for leading Rab5 to the emerging macropinosome (Schnatwinkel et al., 2004). Arf 6, a GTPase protein that is involved in membrane traffic processes, participates in the formation of macropinosomes; recycling Rac1 to the plasma membrane and thereby promoting the formation of a macropinosome (Radhakrishna et al., 1999; Donaldson and Honda, 2005). In addition, ARF6 is also able to affect intracellular traffic to macropinosomes since it affects the Golgi localized ARFs, such as Arf1 that promotes membrane remodeling and endocytic vesicles movement (Donaldson et al., 2009).

Phosphoinositides are involved in cellular processes (e.g., membrane dynamics) and have also been implicated in macropinocytosis. Rac 1 and ARF6 activate the phosphorylation, by phosphatydilinositol 4-phosphate 5-kinase (PI4P5K), of phosphatytilinositol 4-phosphate (PI4P) generating PI(4,5)P2, which is localized in membrane ruffles and in macropinosome cups. PI3 kinase action leads to accumulation of PI(3,4,5)P3 during macropinosome formation and recruits GEFs and GAPs, which present PI(3,4,5)P3 PH domains, thus directing actin polymerization by Rho-GTPase activity, crucial for ruffle formation and macropinosome closure. Rab 5 was also observed to accumulate at the macropinosome after the recruitment of PI(3,4,5)P3 (reviewed by Egami et al., 2014). This GTPase is also involved in actin remodeling, although its precise role in this process is poorly understood (Lanzetti et al., 2004). Using a probe that specifically binds to PI(3,4)P2, transient elevation of this phosphoinositide was shown at the time of cup closure (Welliver and Swanson, 2012; Maekawa et al., 2014). Several pathogens depend on the activation of PI3 Kinase to invade host cells, including viruses (reviewed by Diehle and Schaal, 2013), some bacteria (e.g., *Pseudomonas aeruginosa*, Lovewell et al., 2014), and trypanosomatids (e.g., *Leishmania Mexicana*, Oghumu and Satoskar, 2013), *Leishmania donovani* (Mukherjee et al., 2014), and *Trypanosoma cruzi* (Vieira et al., 2002; Woolsey et al., 2003). However, as this pathway is also crucial for the formation of phagosomes (reviewed by Levin et al., 2015), together with the fact that most groups are verified as having an involvement with this pathway during pathogen entry, suggests a phagocytosis event, rather than a macropinocytosis event, might be responsible. Therefore, macropinocytosis might be more frequent than is currently appreciated because of a lack of investigation of the components that characterize this pathway. In the case of *T. cruzi*, the activation of PI3K pathway was described as the major *T. cruzi'*s host cell entry (Woolsey et al., 2003). In this pathway lysosomes' fusion (essential for parasitophorous vacuole acidification) occurs with a pre formed vacuole and, thus, broke the paradigm that the main route of entry of this protozoan was dependent of lysosome exocytosis to the plasma membrane (Andrews, 1994). The recruitment of PI3K to the forming *Toxoplasma gondii* parasitophorous vacuole was also observed when non-professional phagocyte cell lines were used. The main mechanism of host cell invasion used by *T. gondii* is active penetration, forming a non-fusogenic vacuole (reviewed by Sibley, 2011), but those results indicate that a macropinocytosis event may also occur during *T. gondii* invasion into host cells, since the same group also demonstrates participation of other molecules involved with this process [e.g., ARF6, various phosphoinositides (PIP2, PIP3) and actin cytoskeleton (da Silva et al., 2009)]. Results from different authors using macrophage, a fibroblast cell line or a bladder tumor cell 4934 treated with cytochalasin showed that this drug inhibit but do not block *T. gondii* entry into host cell, so we can suggest that not only phagocytosis but also micropinocytosis could be used by this protozoa (Ryning and Remington, 1978; Silva et al., 1982). In *T cruzi*, macropinocytic cups like structures were visualized using microscopy by Schenkman and Mortara (1992) but any inference was made to a macropinocytosis process, since host cell actin recruitment (essential for macropinocytosis pathway) has been ruled out by the group. In relation to actin, their participation in entry and in the formation of parasitophorous vacuole has been demonstrated quite contradictory, since while some groups exclude their participation (Schenkman and Mortara, 1992) others describe their participation in the entry and VP formation (Vieira et al., 2002; Woolsey et al., 2003; Reignault et al., 2014). Besides, Reignault et al. (2014) demonstrated the formation of an actin belt around the VP in macrophages, which could be related to macropinocytosis (**Figure 2**).

In addition to the Ras superfamily GTPases, macropinocytosis events are also dependent of others protein kinases. The most important of these is the protein kinase activator of p21 (Pak1). Pak1 is a serine threonine kinase type protein that activates Rac1 and Cdc 42, whose function is to regulate the motility and dynamics of the cytoskeleton, which is required during all stages of macropinocytosis (Mercer and Helenius, 2009). Concerning this, using MDCK transfected with constitutively active Rac 1 or Cdc 42, our group has shown that these GTPases participate in the process of *T. cruzi* invasion (Dutra et al., 2005). The group also suggested the participation of Pak1, specific regulators of Pak1, and serine/treonine kinase in the process, supporting the participation of actin filament dynamics during *T. cruzi* invasion. Once activated, Pak1 is relocated to the plasma membrane where it activates a number of effectors required for the formation of ruffling, blebbing and macropinosomes (Mercer and Helenius, 2009). Pak1 is also capable of activating bar proteins (protein 1/Brefeldin A-ADP ribosylated substrate) that are required for closing macropinosomes (Mercer and Helenius, 2009). Protein kinase C (PKC) is another kinase that participates in macropinocytosis. PKC is a Ca^2+^ and diacylglycerol protein serine/threonine kinase that is activated by tyrosine kinase receptor or PI3K, and that after association with the plasma membrane promotes ruffling and the formation of macropinosomes (Mercer and Helenius, 2009). Although the exact function of PKC remains unclear, it is known to be involved in signal transduction and amplification (Ridley and Hall, 1996). In addition to Pak1, PKC, and c-Src protein (protein tyrosine kinase that has a receptor function) also stimulate macropinocytosis (Amyere et al., 2002). Thus, the c-Src receptor works synergistically with tyrosine kinases to further increase macropinosome formation signaling (Donepudi and Resh, 2008). Given the importance of PKC for macropinocytosis, Barrias et al. (2012) used a PKC inhibitor (rotllerin) and a known PKC activator (phorbol 12-myristate 13-acetate - PMA) to demonstrate the participation of this pathway in the internalization processes of trypomastigote and amastigotes of *T. cruzi* by both phagocytic and non-phagocytic host cells. Host cell PKC was also described as essential for internalization of *T. gondii* since this protein is required for infection-induced MAPK activation and production of IL-12, which function as regulators of the innate immune response to T. *gondii* stimuli (Masek et al., 2006).

The use of Na^+^/H^+^ channels inhibitors such as amiloride and EIPA [5-(N-ethyl-N-isopropyl) amiloride] results in a blockage in the formation of ruffling membrane (Dowrick et al., 1993). Regarding this, Barrias et al. (2012) have shown that the using EIPA strongly inhibits the entry of *T. cruzi* into host cells. In many cases the inhibition caused by the use of these inhibitors is the principal tool to study macropinocytosis (Dowrick et al., 1993), however, as there are many events that are cell macropinocytosis-specific, this should not be the only criterion used to classify a macropinocytic event (Ivanov, 2008). Another experimental condition that inhibits macropinocytosis is the depletion of cholesterol (Grimmer et al., 2002). This effect is due to the redistribution of plasma membrane phosphoinositides that affect the location of Rac1, ARF6 and other signaling factors (Grimmer et al., 2002; Kwik et al., 2003). In the case of *T*. *cruzi*, several studies have shown that cholesterol depletion causes a severe inhibition in the parasite's entry into host cells (Barrias et al., 2007; Hissa et al., 2012). The same was observed with with *T. gondii* (Cruz et al., 2014). In all cases, a decrease in the pathogens' entry into the host cells was related to disorganization of membrane microdomain regions, however, we as yet cannot rule out the possibility that depletion of cholesterol is inhibiting the macropinocytosis pathway and thus hampering the entry of parasites. Several proteins are required for the final closure of a macropinosome, allowing this structure to be internalized and gain access to the cytoplasm of the cell. In many cases various classes of myosin (I, II, V, and X) also associate to the closure of the assembling vesicle. The use of inhibitors against these classes of myosins showed that there was a change in curvature during ruffle formation and macropinosome closure. Furthermore, in some cases it was shown that Dynamin, a protein involved in the cleavage of vesicles, participates in this process (Liu et al., 2008). Dynamin has also been described as being responsible for the fission of parasitophorous vacuoles during host cell invasion by *T. cruzi* (Barrias et al., 2012) and *T. gondii* (Caldas et al., 2010), suggesting the use of macropinocytosis in the entry of these pathogens. Macropinocytosis is described as being involved in immunity and infection, as well as being the main pathway used by cells involved in antigen presentation of the class I and class II major histocompatibility complexes (reviewed in Levin et al., 2015). Some studies also associate macropinocytosis with the process of antigen presentation in dendritic cells (Watts, 1997). Macropinocytosis is also described as being involved in the internalization of *Legionella pneumophila*, several viruses (Mercer and Helenius, 2009) and also protozoa. Wanderley et al. (2006) have shown that *L. amazonensis* amastigotes, which expose phosphatidylserine on their surface, are able to induce entry through a macropinocytic process although in the most of the cases *Leishmania* internalization occur using receptor mediated phagocytosis (reviewed by Sibley, 2011). Parasites internalized by this route are localized in loose parasitophorous vacuoles. Recently, Ramos et al. (2014) showed that internalization of different *Leishmania* species by microglia seems to occur by receptor mediated phagocytosis and macropinocytosis, reinforcing the role of this process in antigen presentation. Our group, (Barrias et al., 2012) has shown that macropinocytosis can be used by *T. cruzi* to gain access to the intracellular environment of host cells (Figure 1). Subsequently, Butler et al. (2013) showed that vesicles coated with *T. cruzi* trans-sialidase stimulates a process of “eat me” in epithelial cells in a process similar to macropinocytosis. This conclusion was reached using various approaches, such as showing the participation of Rabankyrin 5, tyrosine kinases, actin, and Pak1 in *T. cruzi* invasion process. Rabankyrin 5 is a PI(3)P-binding Rab 5 effector that can be localized to macropinosomes (Schnatwinkel et al., 2004), and as such can be used as an additional tool to detect macropinosomes. Recently, BoseDasgupta and Pietrs (2014) described that a process that reprograms phagocytosis to macropinocytosis takes place in macrophages during an inflammatory stimuli. This pathway was described as responsible for directing large vacuoles containing a pathogen to be destroyed in lysosomes. The protein involved in this change of endocytosis type pathway is coronin1, whose serine residues are phosphorylated by a protein kinase C after macrophage activation with IFN gamma. Once phosphorylated, coronin1 activates phosphoinositol PI-3-kinase, which is involved, in micropinocytosis, as described above. Therefore, it is tempting to speculate that during the early host infection by *T. cruzi*, when there is an IFN gamma immune response (Ferreira et al., 2014), activated macrophages are reprogrammed to engulf parasites using macropinocytosis. This receptor independent mechanism is more efficient at removing microorganisms from the extracellular space. Regarding *T. cruzi* host cell infection, it is not yet clear how the scenario progresses after entry into host cell of more than one parasite. For cells infected by *T. cruzi* it has been well established that fusion of lysosomes with the parasitophorous vacuole (PV) is necessary for subsequent lysis of the vacuolar membrane, allowing the parasite to enter into contact with the host cell cytoplasm. Parasites that enter host cells using macropinocytosis are directed to lysosomes to be destroyed and antigen processed, possibly limiting the number of live parasite inside the host cell, protecting the host from death (Figure 2). As described above, *T. cruzi* requires an acidic milieu to escape from the PV. Therefore, more studies will be necessary in order to better understand the mechanisms involved in *T. cruzi* entry into a host cell using macropinocytosis and its subsequent fate.

**Figure 1:**
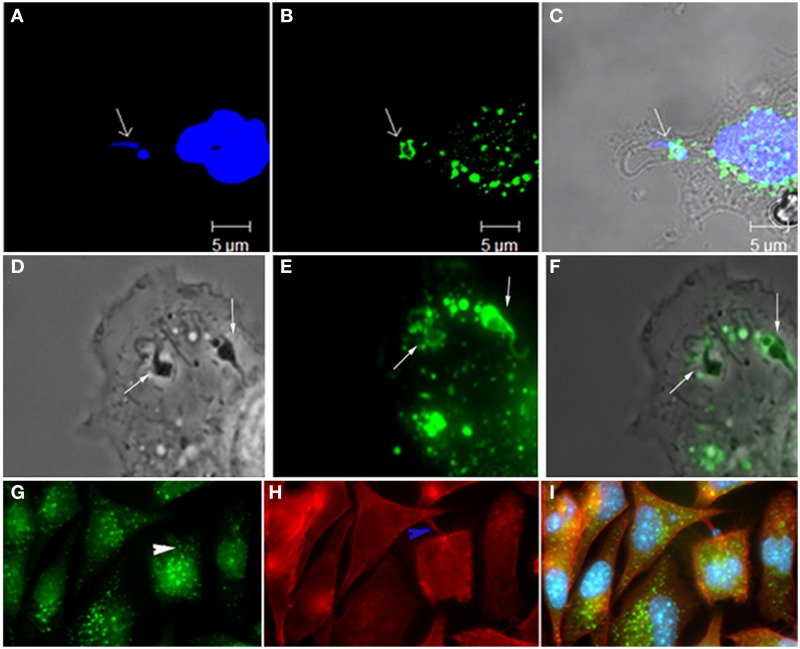
**Participation of Rabankirin 5 (A, DAPI; B, Rabankirin; C, merge), lucifer yellow (D, phase; E, lucifer yellow; F, merge), and actin+rabankirin (G, rabankirin; H, phaloidin Alexa 546, I, merge) in the entry of *T. cruzi* and formation of the parasitophorous vacuole**. These labels are considered of macropinocytosis' markers and thus indicate the participation of this endocytic process in host cell invasion by this protozoan. Images that composes this figure come from Barrias et al. (2012).

**Figure 2:**
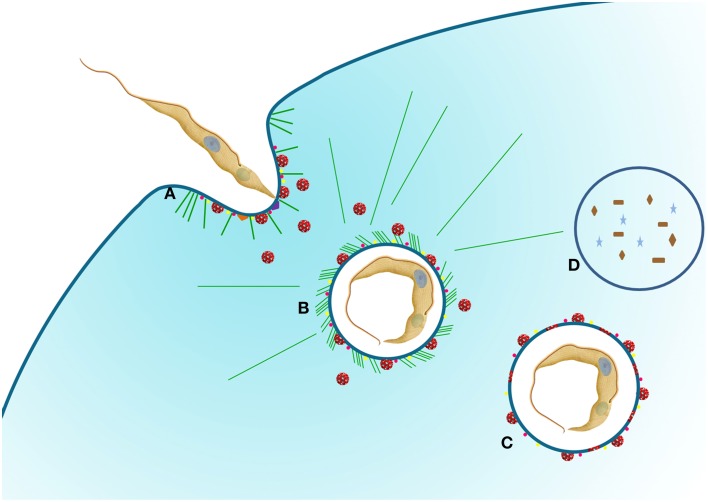
**Schematic representation of *T. cruzi* entry into host cells. (A)** The protozoan adheres to the host cell using different receptors that can stimulate pathways such as PKC (purple triangle) and PI3K (orange triangle). At this moment, the recruitment of lysosomes (red vesicles) to the plasma membrane can occurs. PAK1 (pink dots) and rabankyrin 5 (yellow dots) are also recruited for parasitophorous vacuole (PV), indicating a macropinocytosis pathway. **(B)** With the vacuole formed, lysosomes are still recruited to the PV promoting acidification and labeling with PAK1 and rabankyrin 5 are also present. At this time the parasite can survive and continue the life cycle **(C)** or be degraded **(D)**. Actin filaments (green lines) can be observed. We used *T. cruzi* as an example of protozoa that can use macropinocytosis pathway.

## Conflict of interest statement

The authors declare that the research was conducted in the absence of any commercial or financial relationships that could be construed as a potential conflict of interest.

## References

[B1] Amyere M. Mettlen M. Van Der Smissen P. Platek A. Payrastre B. Veithen A.. (2002). Origin, originality, functions, subversions and molecular signalling of macropinocytosis. Int. J. Med. Microbiol. 291, 487–494. 10.1078/1438-4221-00157 11890548

[B2] Andrews N. W. (1994). From lysosomes into the cytosol: the intracellular pathway of *Trypanosoma cruzi*. Braz. J. Med. Biol. Res. 27, 471–475. 8081267

[B3] Araki N. Johnson M. T. Swanson J. A. (1996). A role for phosphoinositide 3-kinase in the completion of macropinocytosis and phagocytosis by macrophages. J. Cell Biol. 135, 1249–1260. 10.1083/jcb.135.5.1249 8947549 PMC2121091

[B4] Barrias E. S. Dutra J. M. De Souza W. Carvalho T. M. U. (2007). Participation of macrophage membrane rafts in *Trypanosoma cruzi* invasion process. Biochem. Biophys. Res. Commun. 363, 828–834. 10.1016/j.bbrc.2007.09.068 17904520

[B5] Barrias E. S. Reignault L. C. De Souza W. Carvalho T. M. (2012). *Trypanosoma cruzi* uses macropinocytosis as an additional entry pathway into mammalian host cell. Microbes Infect. 14, 1340–1351. 10.1016/j.micinf.2012.08.003 23010292

[B6] Bar-Sagi D. Fernandez A. Feramisco J. R. (1987). Regulation of membrane turnover by ras proteins. Biosci. Rep. 7, 427–434. 10.1007/BF01362505 3315033

[B7] BoseDasgupta S. Pietrs J. (2014). Inflamatory stimuli reprogram macrophage phagocytosis to macropinocytosis for the rapid elimination of pathogens. PLoS Pathogens 10:e1003879. 10.1371/journal.ppat.1003879 24497827 PMC3907376

[B8] Butler C. E. de Carvalho T. M. Grisard E. C. Field R. A. Tyler K. M. (2013). Trans-sialidase stimulates eat me response from epithelial cells. Traffic 4, 853–869. 10.1111/tra.12078 23601193 PMC3770925

[B9] Caldas L. A. Attias M. De Souza W. (2010). Dynamin inhibitor impairs *Toxoplasma gondii* invasion. FEMS Microbiol Lett. 301, 103–108. 10.1111/j.1574-6968.2009.01799.x 19817867

[B10] Cruz K. D. Cruz T. A. Veras de Moraes G. Paredes-Santos T. C. Attias M. de Souza W. (2014). Disruption of lipid rafts interferes with the interaction of *Toxoplasma gondii* with macrophages and epithelial cells. Biomed. Res. Int. 2014:687835. 10.1155/2014/687835 24734239 PMC3964738

[B11] da Silva C. V. da Silva E. A. Cruz M. C. Chavrier P. Mortara R. A. (2009). ARF6, PI3-kinase and host cell actin cytoskeleton in *Toxoplasma gondii* cell invasion. Biochem. Biophys. Res. Commun. 378, 656–661. 10.1016/j.bbrc.2008.11.108 19061866

[B12] Diehle N. Schaal H. (2013). Make yourself at home: viral hijacking of the PI3K/Akt signaling pathway. Viruses 5, 3192–3212. 10.3390/v5123192 24351799 PMC3967167

[B13] Doherty G. J. McMahon H. T. (2009). Mechanisms of endocytosis. Annu. Rev. Biochem. 78, 857–902. 10.1146/annurev.biochem.78.081307.110540 19317650

[B14] Donaldson J. G. Honda A. (2005). Localization and function of Arf family GTPases. Biochem. Soc. Trans. 33, 639–642. 10.1042/BST0330639 16042562

[B15] Donaldson J. G. Porat-Shliom N. Cohen L. A. (2009). Clathrin-independent endocytosis: a unique platform for cell signaling and PM remodeling. Cell Signal. 21, 1–6. 10.1016/j.cellsig.2008.06.020 18647649 PMC2754696

[B16] Donepudi M. Resh M. D. (2008). c-Src trafficking and co-localization with the EGF receptor promotes EGF ligand-independent EGF receptor activation and signaling. Cell Signal. 20, 1359–1367. 10.1016/j.cellsig.2008.03.007 18448311 PMC2459337

[B17] Dowrick P. Kenworthy P. Mccann B. Warn R. (1993). Circular ruffle formation and closure lead to macropinocytosis in hepatocyte growth factor/scatter factor-treated cells. Eur. J. Cell Biol. n61, 44–53. 8223707

[B18] Dutra J. M. Bonilha V. L. De Souza W. Carvalho T. M. (2005). Role of small GTPases in *Trypanosoma cruzi* invasion in MDCK cell lines. Parasitol. Res. 96, 171–177. 10.1007/s00436-005-1333-7 15864650

[B19] Egami Y. Taguchi T. Maekawa M. Arai H. Araki N. (2014). Small GTPases and phosphoinositides in the regulatory mechanisms of macropinosome formation and maturation. Front. Physiol. 5:374. 10.3389/fphys.2014.00374 25324782 PMC4179697

[B20] Ferreira L. R. Frade A. F. Baron M. A. Navarro I. C. Kalil J. Chevillard C.. (2014). Interferon-γ and other inflammatory mediators in cardiomyocyte signaling during Chagas disease cardiomyopathy. World J. Cardiol. 6, 782–790. 10.4330/wjc.v6.i8.782 25228957 PMC4163707

[B21] Fujii M. Kawai K. Egami Y. Araki N. (2013). Dissecting the roles of Rac1 activation and deactivation in macropinocytosis using microscopic photo-manipulation. Sci. Rep. 3:2385 10.1038/srep02385 23924974 PMC3737501

[B22] Grimmer S. Van Deurs B. Sandvig K. (2002). Membrane ruffling and macropinocytosis in A431 cells require cholesterol. J. Cell Sci. 115, 2953–3292. 12082155 10.1242/jcs.115.14.2953

[B23] Hissa B. Duarte J. G. Kelles L. F. Santos F. P. del Puerto H. L. Gazzinelli-Guimarães P. H.. (2012). Membrane cholesterol regulates lysosome-plasma membrane fusion events and modulates *Trypanosoma cruzi* invasion of host cells. PLoS Negl Trop Dis. 6:e1583. 10.1371/journal.pntd.0001583 22479662 PMC3313932

[B24] Hoeller D. Volarevic S. Dikic I. (2005). Compartmentalization of growth factor receptor signalling. Curr. Opin. Cell Biol. 17, 107–111. 10.1016/j.ceb.2005.01.001 15780584

[B25] Ivanov A. I. (2008). Pharmacological inhibition of endocytic pathways: is it specific enough to be useful? Methods Mol. Biol. 440, 15–33. 10.1007/978-1-59745-178-9_2 18369934

[B26] Johannes L. Lamaze C. (2002). Clathrin-dependent or not: is it still the question? Traffic 3, 443–451. 10.1034/j.1600-0854.2002.30701.x 12047552

[B27] Kwik J. Boyle S. Fooksman D. Margolis L. Sheetz M. P. Edidin M. (2003). Membrane cholesterol, lateral mobility, and the phosphatidylinositol 4,5-bisphosphate-dependent organization of cell actin. Proc. Natl. Acad. Sci. U.S.A. 100, 13964–13969. 10.1073/pnas.2336102100 14612561 PMC283529

[B28] Lanzetti L. Di Fiore P. P. (2008). Endocytosis and cancer: an ‘insider’ network with dangerous liaisons. Traffic 9, 2011–2021. 10.1111/j.1600-0854.2008.00816.x 18785924

[B29] Lanzetti L. Palamidessi A. Areces L. Scita G. Di Fiore P. P. (2004). Rab 5 signaling GTPase involved in actin remodeling by receptor tyrosine kinases. Nature 429, 309–314. 10.1038/nature02542 15152255

[B30] Levin R. Grinstein S. Schlam D. (2015). Phosphoinositides in phagocytosis and macropinocytosis. Biochim. Biophys. Acta. 1851, 805–823. 10.1016/j.bbalip.2014.09.005 25238964

[B31] Liu Y. W. Surka M. C. Schroeter T. Lukiyanchuk V. Schmid S. L. (2008). Isoform and splice-variant specific functions of dynamin-2 revealed by analysis of conditional knock-out cells. Mol. Biol. Cell 19, 5347–5359. 10.1091/mbc.E08-08-0890 18923138 PMC2592655

[B32] Lovewell R. R. Patankar Y. R. Berwin B. (2014). Mechanisms of phagocytosis and host clearance of *Pseudomonas aeruginosa*. Am. J. Physiol. Lung Cell Mol. Physiol. 306, L591–L603. 10.1152/ajplung.00335.2013 24464809 PMC4116407

[B33] Maekawa M. Terasaka S. Mochizuki Y. Kawai K. Ikeda Y. Araki N.. (2014). Sequential breakdown of 3-phosphorylated phosphoinositides is essential for the completion of macropinocytosis. Proc. Natl. Acad. Sci. U.S.A. 111, E978–E987. 10.1073/pnas.1311029111 24591580 PMC3964036

[B34] Marsh M. Helenius A. (2006). Virus entry: open sesame. Cell 124, 729–740. 10.1016/j.cell.2006.02.007 16497584 PMC7112260

[B35] Masek K. S. Fiore J. Leitges M. Yan S. F. Freedman B. D. Hunter C. A. (2006). Host cell Ca2+ and protein kinase C regulate innate recognition of *Toxoplasma gondii*. J. Cell Sci. 119(Pt 21), 4565–4573. Erratum in: *J. Cell Sci*. 15, 119, 4789. 10.1242/jcs.03206 17074836

[B36] Mercer J. Helenius A. (2009). Virus entry by macropinocytosis. Nat. Cell Biol. 11, 510–520. 10.1038/ncb0509-510 19404330

[B37] Mukherjee M. Basu Ball W. Das P. K. (2014). *Leishmania donovani* activates SREBP2 to modulate macrophage membrane cholesterol and mitochondrial oxidants for establishment of infection. Int. J. Biochem. Cell Biol. 55C, 196–208. 10.1016/j.biocel.2014.08.019 25218172

[B38] Oghumu S. Satoskar A. R. (2013). PI3K-γ inhibitors in the therapeutic intervention of diseases caused by obligate intracellular pathogens. Commun. Integr. Biol. 6:e23360. 10.4161/cib.23360 23749323 PMC3609848

[B39] Radhakrishna H. AL-Awar O. Khachikian Z. Donaldson J. G. (1999). ARF6 requirement for Rac ruffling suggests a role for membrane trafficking in cortical actin rearrangements. J. Cell Sci. 112, 855–866. 10036235 10.1242/jcs.112.6.855

[B40] Ramos P. K. Brito Mde V. Silveira F. T. Salgado C. G. De Souza W. Picanço-Diniz C. W.. (2014). *In vitro* cytokines profile and ultrastructural changes of microglia and macrophages following interaction with *Leishmania*. Parasitology 141, 1052–1063. 10.1017/S0031182014000274 24717447

[B41] Reignault L. C. Barrias E. S. Soares Medeiros L. C. de Souza W. de Carvalho T. M. (2014). Structures containing galectin-3 are recruited to the parasitophorous vacuole containing *Trypanosoma cruzi* in mouse peritoneal macrophages. Parasitol Res. 113, 2323–2333. 10.1007/s00436-014-3887-8 24760627

[B42] Ridley A. J. Hall A. (1996). Distinct patterns of actin organization regulated by the small GTP-binding proteins Rac and Rho. Cold Spring Harb. Symp. Quant. Biol. 57, 661–671. 10.1101/SQB.1992.057.01.072 1339704

[B43] Ryning F. W. Remington J. S. (1978). Effect of cytochalasin D on *Toxoplasma gondii* cell entry. Infect. Immun. 20, 739–743. 669821 10.1128/iai.20.3.739-743.1978 PMC421921

[B44] Schenkman S. Mortara R. A. (1992). HeLa cells extend and internalize pseudopodia during active invasion by *Trypanosoma cruzi* trypomastigotes. J. Cell Sci. 101(Pt 4), 895–905. 1527184 10.1242/jcs.101.4.895

[B45] Schnatwinkel C. Christoforidis S. Lindsay M. R. Uttenweiler-Joseph S. Wilm M. Parton R. G.. (2004). The Rab5 effector Rabankyrin-5 regulates and coordinates different endocytic mechanisms. PLoS Biol. 9:E261. 10.1371/journal.pbio.0020261 15328530 PMC514490

[B46] Sibley L. D. (2011). Invasion and intracellular survival by protozoan parasites. Immunol. Rev. 240, 72–91. 10.1111/j.1600-065X.2010.00990.x 21349087 PMC3697736

[B47] Silva S. R. Meirelles S. S. De Souza W. J. (1982). Mechanism of entry of *Toxoplasma gondii* into vertebrate cells. Submicrosc. Cytol. 14, 471–482. 7175984

[B48] Veiga E. Cossart P. (2006). The role of clathrin-dependent endocytosis in bacterial internalization. Trends Cell Biol. 16, 499–504. 10.1016/j.tcb.2006.08.005 16962776 PMC7126422

[B49] Vieira M. Dutra J. M. Carvalho T. M. Cunha-e-Silva N. L. Souto-Padron T. De Souza W. (2002). Cellular signalling during the macrophage invasion by *Trypanosoma cruzi*. Histochem. Cell Biol. 118, 491–500. 10.1007/s00418-002-0477-0 12483314

[B50] Wanderley J. L. Moreira M. E. Benjamin A. Bonomo A. C. Barcinski M. A. (2006). Mimicry of apoptotic cells by exposing phosphatidylserine participates in the establishment of amastigotes of *Leishmania* (*L*) *amazonensis* in mammalian hosts. J Immunol. 176, 1834–1839. 10.4049/jimmunol.176.3.1834 16424214

[B51] Watts C. (1997). Capture and processing of exogenous antigens for presentation on MHC molecules. Annu. Rev. Immunol. 15, 821–850. 10.1146/annurev.immunol.15.1.821 9143708

[B52] Welliver T. P. Swanson J. A. (2012). A growth factor signaling cascade confined to circular ruffles in macrophages. Biol. Open 1, 754–760. 10.1242/bio.20121784 23213469 PMC3507227

[B53] Woolsey A. M. Sunwoo L. Petersen C. A. Brachmann S. M. Cantley L. C. Burleigh B. A. (2003). Novel PI 3-kinase-dependent mechanisms of trypanosome invasion and vacuole maturation. J. Cell Sci. 116, 3611–3622. 10.1242/jcs.00666 12876217

